# Learning Outcomes of High-fidelity versus Table-Top Simulation in Undergraduate Emergency Medicine Education: Prospective, Randomized, Crossover-Controlled Study

**DOI:** 10.5811/westjem.2021.12.53926

**Published:** 2022-01-03

**Authors:** Joseph Offenbacher, Alexander Petti, Han Xu, Michael Levine, Mallika Manyapu, Debayan Guha, Maxim Quint, Andrew Chertoff, Andrew Restivo, Benjamin W. Friedman, Joshua Silverberg

**Affiliations:** Albert Einstein College of Medicine, Department of Emergency Medicine at the Jacobi and Montefiore Hospitals, Bronx, New York

## Abstract

**Introduction:**

Over the last several decades simulation, in both graduate and undergraduate emergency medicine education, has continued to develop as a leading and highly effective teaching modality. Limited research exists to evaluate the efficacy of low-fidelity (table-top) simulation, as compared to high-fidelity standards, as it relates to medical knowledge learning outcomes. We sought to assess the efficacy of a low-fidelity simulation modality in undergraduate emergency medicine education, based on quantitative medical knowledge learning outcomes.

**Methods:**

A prospective, randomized, crossover-control study comparing objective medical knowledge learning outcomes between simulation modalities. Analysis was designed to evaluate for the statistical equivalence of learning outcomes between the two cohorts. This was done by comparing a calculated 95% confidence interval (CI) around the mean difference in post-test scores, between experimental and control modalities, to a pre-established equivalence margin.

**Results:**

Primary outcomes evaluating student performance on post-test examinations demonstrated a total cohort CI (95% CI, -0.22 and 0.68). Additional course-subject subgroup analysis demonstrated non-inferior CIs with: Shortness of Breath (95% CI, −0.35 and 1.27); Chest Pain (95% CI, −0.53 and.94); Abdominal Pain (95% CI, −0.88 and 1.17); Cardiovascular Shock (95% CI, −0.04 and 1.29). Secondary outcome analysis was done to evaluate medical knowledge acquisition by comparing the difference in pre and post-test examination between the cohorts. CI of the full cohort ranged from (95% CI, −0.14 and 0.96).

**Conclusion:**

The student’s performance on quantitative medical-knowledge assessment was equivalent between the high-fidelity control and low-fidelity experimental simulation groups. Analysis of knowledge acquisition between the two groups also demonstrated statistical equivalence.

## INTRODUCTION

Over the last several decades simulation has continued to develop as a highly effective teaching modality used in a wide range of settings.^1^,^2^ In emergency medicine education the rapid evolution of simulation has relied heavily on cutting-edge technology, with increased levels of fidelity, as well as advanced modality-specific training programs such as post graduate fellowships.^3^ Increased recognition of the potential impact of simulation in emergency medicine education has grown in the wake of the academic challenges that followed the SARS-COV-2 (COVID-19) pandemic.^4^

In spite of the increasing utilization of simulation in emergency medicine education, significant challenges have persisted.^5^ These include the need for technically skilled operators, simulation trained educators, and substantial material resources.^6^–^8^ To date, the limited existing data has focused heavily on high-fidelity simulation for teaching both medical knowledge and clinical skills.^9^–^12^ Consequently, the integration of simulation into emergency medicine clerkship programs has remained selective, representing a secondary didactic adjunct at the undergraduate level.^13^ In response to these challenges, undergraduate emergency medicine educators have expressed significant interest in the use of low-fidelity (table-top) simulation experiences, despite the lack of outcomes-based research.^14^,^15^

During the 2019 academic year we looked to assess the efficacy of low-fidelity simulation modalities in undergraduate emergency medicine education, and conducted a randomized crossover study comparing a low-fidelity experimental model to a high-fidelity simulation control group.^16^ The primary outcome was medical knowledge acquisition measured by standardized multiple-choice examinations at the end of the one-month clerkship. As the efficacy of high-fidelity simulation control has been well established, our study was designed to assess for statistical equivalence of the experimental low-fidelity modality.

## METHODS

### Setting

The study was conducted in a large urban medical college, where emergency medicine holds full departmental status, with robust undergraduate (UGME) and residency (GME) training programs. Medical students and residents rotate through a Level 1 urban trauma center and referral teaching hospitals. The department offers a four-week clerkship featuring low-fidelity case-based simulation clerkship curriculum inaugurated during the 2018 academic year. Its medical knowledge content is in line with generally accepted national standards set forth by Council of Residency Directors in Emergency Medicine (CORD) and Clerkship Directors in Emergency Medicine (CDEM) guidelines and includes the subjects of: chest pain (CP), shortness of breath (SB), abdominal pain (AP) and cardiovascular shock (CS).

The experimental, low-fidelity, simulation sessions utilized teddy bears as patient models through which participating students interacted with cases. The control high-fidelity simulation was conducted in, the on-campus, Health and Hospitals Institute for Medical Simulation and Advanced Learning (IMSAL) on a Laerdal SimMan®3G mannequin, with residency simulation faculty and additional technical support staff on site, in one of the center’s high-fidelity resuscitation rooms.

**Population Health Research Capsule**

What do we already know about this issue?

*Although emergency medicine has long embraced simulation, the challenges associated with offering high-fidelity experiences remains a significant barrier to widespread implementation*.

What was the research question?

*What is the efficacy of low-fidelity simulation in undergraduate emergency medicine education?*

What was the major finding of the study?

*Low and high fidelity simulation modalities are equivalent when comparing medical-knowledge learning outcomes*.

How does this improve population health?

*Our study provides some of the first data to support low-fidelity simulation as an equivalent modality, to high-fidelity models, as it pertains to medical-knowledge learning outcomes*.

Case-based teaching points for each of the four topics, as well as teaching formats, remained unchanged for the entire 2019 academic year regardless of study assignment and included an initial oral board style case simulation, a clinical knowledge debrief discussion and a summative simulation exercise. As such session structure remained consistent between control and experimental modalities. Other than intrinsic differences of the two modalities, efforts were made to control for all other variables including session duration, identical learning points regardless of learning modality and consistency amongst a small group of educators. Over the course of each clerkship cohort period, all participating students were randomly assigned to participate in two experimental and two control didactic sessions. All students were exposed to all teaching points through either the experimental or control simulation modality.

### Study Design and Population

We used a randomized, crossover design to control for confounders related to the course subject content and individual participants. The 2019 academic year consisted of six clerkship cohorts, designated as either ‘A’ or ‘B,’ totaling fifty-five students. The randomization to determine assignment was performed in May, 2019 and consisted of a coin toss. We randomized the first topic of the first cohort to either experimental or control modality and determined that subsequent topic and subsequent cohorts would alternate topics. (Table 1)

**Figure 1:**
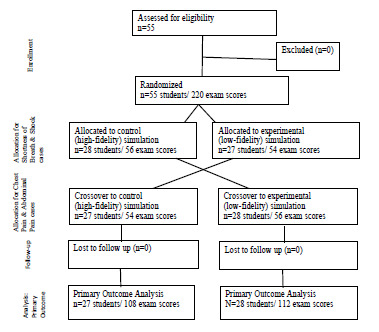
A flowchart of the study design for randomized crossover study of high- versus low-fidelity simulation.

Ultimately, each student participated in two topics taught via the experimental and two topics taught via the control modalities. This crossover design allowed each student to serve as their own control while also controlling for variability related to the specific content of each subject being taught. (Figure 1) Over the course of the study, each topic was taught by each modality an equal number of times. The study was designed for all students, in a given cohort, to experience each of the four areas of content via the same learning modality, with all participating students having the same number of exposures to the control and experimental learning modalities.

All fourth-year medical students in the department’s emergency medicine clerkship were eligible for inclusion in the research study. All students signed a formal consent for participation in research, but were blinded to the study’s objectives and hypothesis. The study had no formal exclusion criteria other than each student’s ability to decide not to participate in the research study.

Although the program’s simulation experience was mandatory for all clerkship participants, their participation in the study was optional. Students were informed, prior to the start of their participation in the course’s educational activities, that their clerkship evaluation would not be affected by their participation in the research study and that their performance on the research study’s activities had no impact on their clerkship evaluation. The university’s institutional review board (IRB) granted the study an educational exemption.

### Outcome Measures

We chose student performance on a summative multiple choice question exam as the study’s primary outcome due to the important role of medical student clerkships in transmitting foundational medical knowledge.^17^ This represents an intermediate level on the Kirkpatrick hierarchy.^18^ We created a collection of forty multiple choice questions, which evaluated the student’s knowledge of the curriculum’s forty discrete teaching points. The forty teaching points and corresponding questions were evenly distributed among the four didactic topics. A ten-question pre-test was given prior to each of the four didactic sessions (totaling 40 test questions per student). All forty questions, which were incorporated into the student’s final course exam, served as the study’s post-test. Student examination performance was defined as the number and percentage of correct responses out of the total number of examination questions.

The primary outcome compared the students’ performance on post-test examinations between the control and experimental cohorts. We performed this analysis for the entire forty question test as well as sub-group analysis for each of the four specific subject topics. The secondary outcome was knowledge acquisition, defined as the magnitude of changes in score between pre and post-test examinations. For this analysis, only post-test data that had a completed corresponding pretest was eligible for inclusion.

We calculated the sample size via the Rollin Brant calculator (https://www.stat.ubc.ca/~rollin/stats/ssize/n2.html) based on the following assumptions: a Standard of Deviation of 5.8; Mean of Group 1=85.9; Mean of Group 2=92.0, an alpha of 0.05 and a beta of 0.2. Assumptions were based on examination scores from prior years. The calculation showed a needed sample size of at least 15 participants in each study arm. The study was thus sufficiently powered to analyze both the primary and secondary outcomes.

### Data Analysis

We used an equivalency analysis.^19^ We assessed equivalence by determining whether the between-group difference and the associated 95% confidence interval (CI) fell entirely within a pre-stablished *equivalence margin* (Δ).^20^,^21^,^22^,^23^

The data from the results of the pre and post-tests were analyzed with Microsoft Excel 365 (Version 1905; Microsoft Office, Redmond, Washington), https://www.socscistatistics.com and R-Studio (Version 1.1.414 – © 2009–2018). Each exam consisted of ten questions with a total value of ten points reflecting a single point earned for each correct question. As such, we established the equivalence margin to be +/− one point (− 1 to 1).

To evaluate the study’s primary outcome, we sought to determine if there was equivalence on post-test performance between the control and experimental groups. First, we calculated the 95% CI around the mean difference in post-test scores between experimental and control for each student. Second, we examined if the 95% CI of this mean difference fell within our pre-established *equivalence margin*. If the 95% CI of the mean difference between the control and experimental groups fell within the equivalence margin, we rejected the null hypothesis that there was a difference between the control and experimental modalities.

Our secondary outcome was to determine if there was a difference between the magnitude of improvement, from pre to post-test examinations, between the two study groups. Differences between pre and post-test performance was calculated by subtracting paired pre-scores and post-scores for each participant in the experimental and control groups respectively. We again calculated the 95% CI around the difference of means for each group pair. As with the primary outcome findings, confidence intervals falling within the equivalence margin demonstrated equivalence between the control and experimental modalities.

## RESULTS

All fifty-five (n=55) students completed the post-test examination and were included in the primary analysis. Two participants were excluded from the secondary analysis due to missing all four pretests. Four additional discrete paired test scores were excluded because four participants missed a single corresponding pretest. In total, 208 scores from fifty-three participants were included in the secondary analysis.

**Figure 2:**
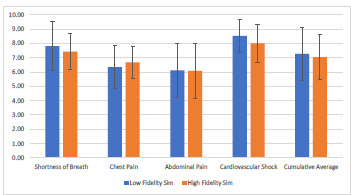
Post-test examination scores of high- versus low-fidelity simulation. *sim*, simulation.

**Figure 3:**
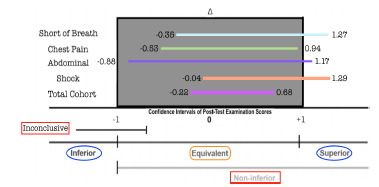
Analysis of post-test examination scores. X- axis depicts confidence intervals (CI) of post-test examination scores with an equivalence margin (Δ) ranging from -1 to 1. Y- axis depicts individual course subject subgroup analysis for, shortness of breath (SB), chest pain (CP), abdominal pain (AP), cardiovascular shock (CS) and mean outcome of the total cohort (TC). Boxed term in orange indicates margins of Statistical Equivalence (representing the study’s primary outcome measure). Boxed terms in red represent graph legend of possible statistical data outcomes including Non-inferiority and Inconclusive. Boxed terms in blue represent graph legend of possible statistical data outcomes including superiority and inferiority.

**Figure 4:**
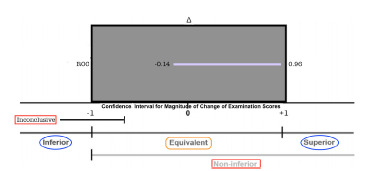
Analysis of magnitude of change of pre- and post- test exam scores. X- axis depicts confidence intervals (CI) of magnitude of change between pretest and post test scores falling withing the pre-set equivalence margin (Δ), ranging from -1 to 1. Y- axis depicts category of magnitude of change between pretest and post test scores of the entire cohort. Boxed term in orange indicates margins of Statistical Equivalence (representing the study’s primary outcome measure). Boxed terms in red represent graph legend of possible statistical data outcomes including Non-inferiority and Inconclusive. Boxed terms in blue represent graph legend of possible statistical data outcomes including superiority and inferiority.

The mean post-test scores for the low-fidelity experimental cohort were: SB- 7.9/10; CP-6.4/10; AP-6.2/10; CS-8.6/10. Across all subjects, the mean post test score for the low-fidelity cohort was 7.3/10. The mean post-test scores for the high-fidelity control cohort were: SB- 7.4/10; CP-6.7/10; AP-6.1/10; CS-7.9/10. Across all subjects, the mean post test score for the high-fidelity cohort was 7.0/10. (Figure 2) Calculated the 95% CI around the difference of means for the cohort’s total score was (95% CI, −0.22 and 0.68). Subject specific CIs were as follows: SB (95% CI, −0.35 and 1.27); CP (95% CI, −0.53 and 0.94); AP (95% CI, −0.88 and 1.17); CS (95% CI, −0.04 and 1.29). (Figure 3) The secondary outcome, considering the difference between groups in magnitude of improvement from pre to post-test examination, was (95% CI, −0.14 and 0.96). (Figure 4)

## DISCUSSION

Our study sought to assess the efficacy of a low-fidelity simulation modality in undergraduate emergency medicine education, based on quantitative medical knowledge learning outcomes.^24^,^25^ These data demonstrated that medical education learning outcomes were equivalent between high-fidelity and low-fidelity cohorts across all topics and within specific topics. Similarly, knowledge gain between the study arms was equivalent.

Although emergency medicine has long embraced simulation the challenges associated with offering high-fidelity experiences remains a significant barrier to widespread implementation. More recently, educators have looked to overcome these challenges in order to integrate simulation into their educational offerings especially in the context of academic challenges related to the SARS-COV-2 pandemic.^26^,^27^ Emergency medicine education, in particular, has faced significant challenges as a result of the high personal risks of infection and illnesses in the emergency department, the heavy emphasis on the medical student clerkship in evaluating prospective applicants, and the central role of clinical training for both GME and UGME emergency medicine training.^28^,^29^

Our study provides some of the first randomized, controlled data to support low-fidelity simulation as an equivalent modality to more traditionally accepted high-fidelity models, as it pertains to medical-knowledge learning outcomes. These findings support existing evidence on the efficacy of simulation as a learning modality, while at the same time challenging the notion that level of fidelity correlates to improved learning outcomes. Our data suggests that simulation programing can be an effective learning modality even when resources for higher levels of fidelity are not available. Future research studies would help to better characterize these findings and extend them to other learning sectors targeted by simulation, such as their impact on clinical outcomes.

## LIMITATIONS

Our study was conducted in a single academic center over the course of a single academic year. Both the control and interventional sessions were taught by members of the research team who were not blinded to the study or its objectives. Although pre-test data was incorporated into secondary outcome analysis, baseline knowledge characteristics of the participating cohorts may have been variable. Despite controlling for this variable, subgroup analysis was not conducted to evaluate learning outcomes as a reflection of time, such that it is not clear how participating in cohorts later in the academic year impacted outcomes. The study was only designed to address medical knowledge learning outcomes. It cannot comment on the relative efficacy of the two modalities with regard to clinical outcomes.

## CONCLUSIONS

We conducted a randomized, crossover-controlled study assessing the equivalence in learning outcomes of high-fidelity and low-fidelity simulation modalities in undergraduate emergency medicine education. Findings showed that the student’s performance on quantitative medical-knowledge assessment was equivalent between the control and experimental groups. Furthermore, analysis of knowledge acquisition between the two groups also demonstrated statistical equivalence.

## Figures and Tables

**Table 1:** Cohort configurations.

Cohort #	1	2	3	4	5	6
Number of students	8	10	12	9	8	8
Configuration*	A**	B	A	B	A	B
Subjects via experimental modality	CP/AP	SB/CS	CP/AP	SB/CS	CP/AP	SB/CS
Subjects via control						
Modality	SB/CS	CP/AP	SB/CS	CP/AP	SB/CS	CP/AP

* Configuration ‘A’ cohorts participated in the Chest Pain (CP) and Abdominal Pain (AP) content sessions using the experimental learning modality, and the Shortness of Breath (SB) and Cardiovascular Shock (CS) content sessions via the control modality. Configuration ‘B’ cohorts participated in the CP and AP content sessions using the control learning modality, and the SB and CS content sessions via the experimental modality.

** Prior to the start of the academic year, cohort number on 1 was randomized (via a non-biased coin toss) to the ‘A’ configuration. Following the initial randomization of the first cohort, all subsequent cohorts strictly adhered to a pre-established rotational configuration. *CP*, chest pain; *AP*, abdominal pain; *SB*, shortness of breath; *CS*, cardiovascular shock.
